# Outcome of Hospitalized Cancer Patients with Hypernatremia: A Retrospective Case-Control Study

**DOI:** 10.3390/curroncol29110693

**Published:** 2022-11-16

**Authors:** Jessica del Rio, Martin Buess

**Affiliations:** 1 Faculty of Medicine, Basel University, 4001 Basel, Switzerland; 2 Faculty of Medicine, Medical Oncology, St. Claraspital and Basel University, 4001 Basel, Switzerland

**Keywords:** hypernatremia, cancer, prognosis

## Abstract

Hypernatremia (>145 mmol/L) is a relatively rare event, and the data regarding its role in the outcome of inpatients on an oncology ward are weak. The aim of this study was to describe the prevalence, prognosis, and outcome of hospitalized cancer patients with hypernatremia. We performed a retrospective case-control study of data obtained from inpatients with a solid tumor at the St. Claraspital, Basel, Switzerland, who were admitted between 2017 and 2020. The primary endpoint was overall survival. Hypernatremia was found in 93 (3.16%) of 2945 inpatients bearing cancer or lymphoma. From 991 eligible normonatremic control patients, 93 were matched according to diagnosis, age, and sex. The median overall survival time (OS) of patients with hypernatremia was 1.5 months compared to 11.7 months of the normonatremic controls (HR 2.69, 95% CI 1.85–3.90, *p* < 0.0001). OS of patients with irreversible compared to reversible hypernatremia was significantly shorter (23 versus 88 days, HR 4.0, 95% CI 2.04–7.70, *p* < 0.0001). The length of hospital stay was significantly longer for the hypernatremic than for the normonatremic group (*p* < 0.0001). Significantly more patients with hypernatremia died in the hospital (30.1% versus 8.6%, *p* < 0.001). These results suggest hypernatremia to be associated with an unfavorable outcome and a very short OS.

## 1. Introduction

Sodium is the most abundant cation in the extracellular space, where it contributes significantly to the maintenance of the extracellular fluid compartment and the osmotic pressure [1]. Under normal conditions, the plasma osmolality is maintained at approximately 280–290 mOsm/kg H_2_O. From as little as a 1% deviation, compensation mechanisms are activated. When osmotic receptors in the hypothalamus notice a rise in osmotic pressure, e.g., in the case of hypernatremia, the hypothalamus activates a thirst response, and the secretion of vasopressin is stimulated, leading to renal tubular water reabsorption [2]. In addition, baroreceptors in the vascular system can detect even the smallest changes in volume through a change in wall tension and thus detect any alteration in sodium content in the extracellular fluid compartment and adjust sodium excretion accordingly [3]. Under physiological conditions, the plasma sodium content is regulated within a narrow range between 135 and 145 mmol/L. Hypernatremia occurs when the plasma sodium concentration exceeds 145 mmol/L [4].

In elderly people and patients with severe diseases, e.g., oncological diseases and intensive therapies, the feeling of thirst is often disturbed, and consequently, the water intake is insufficient [5]. Clinically, hypernatremia manifests itself mainly in the acute form or with neurological symptoms at sodium levels of >160 mmol/L. The severity of the symptoms depends on the time of development, the duration, and the extent of the hypernatremia. Chronic hypernatremia is often less symptomatic [4]. In contrast to children, adults often show rather mild symptoms of hypernatremia [1]. Hypernatremic patients who become symptomatic present with lethargy, weakness, and irritability. They may develop convulsions, seizures, or even coma [6,7]. Oncological patients are particularly vulnerable to electrolyte disturbances due to chemotherapies and their side effects (e.g., nephrotoxicity, vomiting, and diarrhea) [6]. There are still no clear treatment guidelines. Some studies warn against correcting too quickly, others too slowly. Both are associated with increased mortality [8,9]. For the treatment strategy, it is important to discriminate between acute and chronic hypernatremia, as described in the algorithm by Liamis et al. [7].

Hyponatremia (serum sodium <135 mmol/L) is common and well-described in cancer patients [10]. Hypernatremia occurs infrequently, and the data for inpatients on an oncology ward are weak. Our own unsystematic observation suggests that hypernatremia occurs mainly in critically ill patients and is associated with a very poor prognosis. This observation is supported by a study that showed increased morbidity and mortality (5.2% versus 1.3%) in surgical patients with even low levels of hypernatremia compared to patients with normonatremia [8]. A second study showed that unselected patients with hypernatremia had a significantly worse outcome with increased in-hospital mortality and increased resource requirements [9]. A study from the M.D. Anderson Cancer Center described a five-fold increase in mortality and length of hospital stay in hematological and oncological patients with hypernatremia [5]. This study included oncological and hematological in-patients with a high proportion of leukemias from the normal ward and the intensive care unit. We could not find any data in the literature that could directly be transferred to a normal oncology ward with a mixed patient population bearing solid tumors (cancers and lymphoma). Cancer patients with hypernatremia assigned to hospice care exhibited a dismal prognosis of 1–4 weeks [11,12]. One could speculate whether hypernatremia, if associated with such a short survival, could serve as a factor for the decision to forgo the antineoplastic therapy in favor of a pure “best supportive care”-strategy.

The aim of this study was to describe the prevalence of hypernatremia, the clinical outcome of hypernatremia, and the duration of hospital stay and compare it with normonatremic patients in the oncology ward in the St. Claraspital, Basel.

## 2. Materials and Methods

### 2.1. Participants and Data Collection

We performed a single-center case-control study to analyze the outcome of oncological patients with hypernatremia who were hospitalized between 1 January 2017 and 31 December 2020 at the St. Claraspital in Basel. 

Patients were retrospectively identified through the hospital’s administrative- and laboratory database CGM VT (CompuGroup Medical SE & Co. KGaA, Koblenz, Germany). These two databases contained all hospitalized patients from the oncology wards, including diagnosis and all serum sodium measurements. Serum sodium was measured using cobas^®^ pro from Roche/Hitachi with the ISE method (ion selective electrodes).

The electronic health records (CGM Phoenix, CompuGroup Medical SE & Co. KGaA, Koblenz, Germany) provided the following data: gender, age, date of death or day the patient was last known alive, date of the first laboratory-confirmed hypernatremia, duration of hospital stay, vitals, co-morbidities (Table S1), status at hospital discharge, stage of the underlying oncological disease and the presence of brain metastases. The covariates for the matching were main cancer diagnosis, age, and sex. 

Data were collected until 31 December 2021 to ensure a minimum follow-up time of 12 months for all patients. For the normonatremic group the examination day was the day of admission on the last hospital stay, and for the hypernatremic group, it was the day the hypernatremia was measured during the last hospital stay.

### 2.2. Endpoints

The primary endpoint of this case-control study was overall survival, measured from the time of the first occurrence of hypernatremia (hypernatremic group) or the day of the last hospital admission (normonatremic group) until the death of the cohort of patients with hypernatremia compared to the cohort with normonatremia treated in the oncology ward of St. Claraspital between 2017 and 2020. Patients who were lost to follow-up were censored at the date of their last contact. Secondary endpoints are the following: The frequency of hypernatremia in hospitalized patients in oncology ward, the frequency of hypernatremia at hospital admission versus during the hospitalization, the frequency of correctable (sodium value within a normal range after detection of a hypernatremic sodium value) versus non-correctable hypernatremia (no documented sodium value within normal range after hypernatremia), the length of hospital stay (mean and median) of patients with hypernatremia versus patients with normonatremia, the in-hospital-mortality, the 90-day mortality and functional status at discharge (discharge home vs. rehabilitation vs. hospice care).

### 2.3. Statistical Analysis

Observed variables were tabulated using Microsoft^®^ Excel^®^, version 2007. Continuous variables are expressed as median, mean, and range, and categorical variables as percentages and numbers. Continuous variables were compared using a two-tailed *t*-test and Chi-squared or Fisher’s exact test for categorical variables. Kaplan–Meier method was used to estimate overall survival. Cox proportional hazard ratio and the log-rank test were performed to compare the survival curves. The reverse Kaplan–Meier method was used to calculate the median follow-up time. Statistical analyses were performed using R/RStudio, version 4.1.3 (RStudio: Integrated Development for R. RStudio, PBC, Boston, MA, USA) and GraphPad Prism, version 9.3.1 for Windows, (GraphPad Software, San Diego, CA, USA). *p*-values of an alpha level of <0.05 were considered significant.

## 3. Results

### 3.1. Patients Characteristics

**Figure 1:**
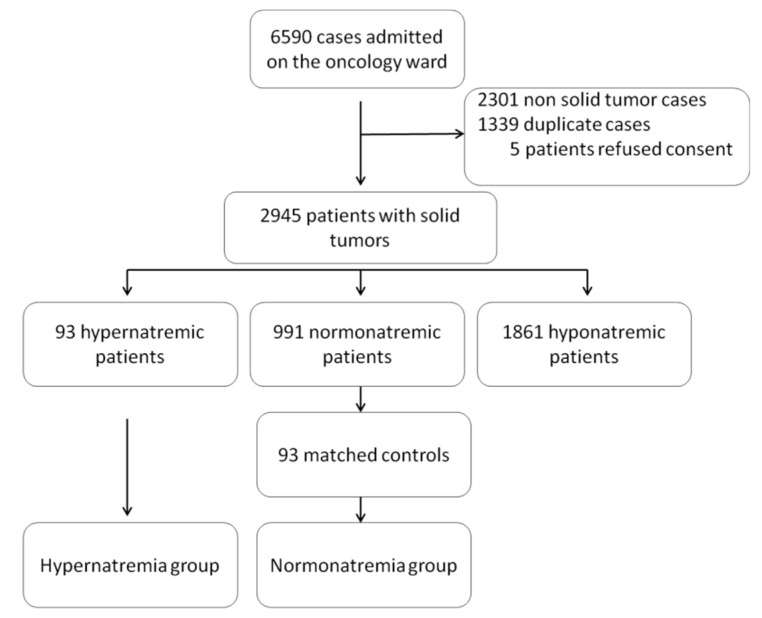
Patients Enrolment. Of 6590 patient cases treated on the oncology ward, 3645 were excluded. Of 2945 patients with a solid tumor, 93 patients presented with hypernatremia and were matched with 93 patients from a cohort of 991 strictly normonatremic patients.

Between 1 January 2017, and 31 December 2020, 6590 patients cases admitted to the oncology wards of the St. Claraspital were screened. Figure 1 gives an overview of the sampling procedure. The following cases were excluded from further analysis: 1339 cases without a solid tumor (cancer or lymphoma), 2301 duplicate cases, and 5 patients who refused data disclosure. Among 2945 patients, 93 (3.2%) presented with hypernatremia with at least one serum sodium value of >145 mmol/L and no value <135 mmol/L. In total, 1861 patients showed hyponatremia with at least one serum sodium value <135 mmol/L and were excluded from further analysis. In total, 991 patients who were strictly normonatremic without any serum sodium value of >145 mmol/L and <135 mmol/L were eligible to be matched controls. From this group, 93 were matched with the hypernatremic patients for cancer type, age, and sex.

A total of 186 patients were included in the study: 93 patients with hypernatremia and 93 matched controls with strictly normonatremic values. Due to the main matching criteria, the type of cancer and gender were equally distributed, with 52 males and 41 females in each group. For both groups, the median age was 77 years (range of 46–93 years and 43–90 years, respectively). The median sodium level on the observation day was significantly different between the two groups, with 147 mmo/L (range of 146–160 mmol/L) for the hypernatremic group and 139 mmol/L (range of 135–146 mmol/L) for the normonatremic group (*p* < 0.00001). The hypernatremic state lasted three days on average. The patients’ baseline characteristics are presented in Table 1. In the study population, lung cancer (30%) was the most common type of tumor, followed by prostate (22%) and esophageal cancer (18%). The complete list of tumor entities is given in Figure S1. All patients exhibited between one and six co-morbidities, except for one patient in the hypernatremic group and nine patients in the normonatremic group, who did not have any relevant diseases other than their underlying cancer. For both groups, the most common co-morbidity was cardiovascular disease (86% in the hypernatremic group vs. 66% in the normonatremic group), followed by kidney disease (49% vs. 27%) and endocrine disorders (44% vs. 30%). Three out of six co-morbidities measured were significantly different between the two groups: cardiovascular disease (*p* = 0.001), kidney disease (*p* = 0.002), and previous malignancies (*p* = 0.014) (Table S2). Most patients had an advanced tumor stage (≥III) on the examination day. Comparing the two groups, the hypernatremic patients showed significantly more advanced stages (stage III and IV) compared to the normornatremic patients (*p* = 0.011), while the normonatremic patients exhibited more early stages (stage I and II) (*p* = 0.002) (Table S3).

### 3.2. Overall Survival in Hypernatremic and Normonatremic Patients

**Figure 2:**
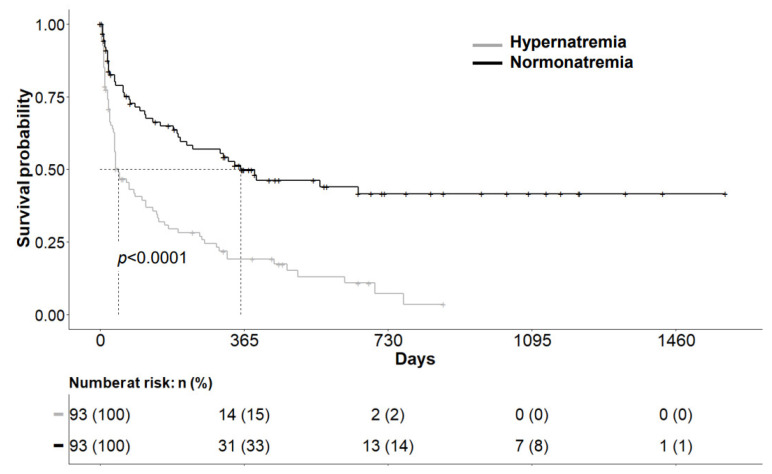
Kaplan–Meier analysis of Overall Survival: Log rank test was performed to compare the survival curves, which showed a significantly different survival (*p* < 0.0001). Median survival time of the hypernatremic group was 1.5 months compared to 11.7 months for the normonatremic group.

The primary endpoint of this study was overall survival (OS). The median follow-up time was 18 months. The survival analysis showed a significantly shorter OS for the hypernatremic patients compared to the normonatremic patients (HR 0.38, 95% CI, 0.26–0.56, *p* < 0.0001). The median OS was 1.5 months for the hypernatremic group compared to 11.7 months for the normonatremic group (Figure 2). The whole cohort of hypernatremic and normonatremic patients showed an OS rate at 12 months of 34% (95% CI, 0.28–0.42). The hypernatremic group had an OS rate at 12 months of 19.1% (95% CI, 0.12–0.30) compared to 49.8% (95% CI, 0.40–0.62) for normonatremic patients.

### 3.3. Frequency of Hypernatremia Depending on the Underlying Malignancy 

**Figure 3:**
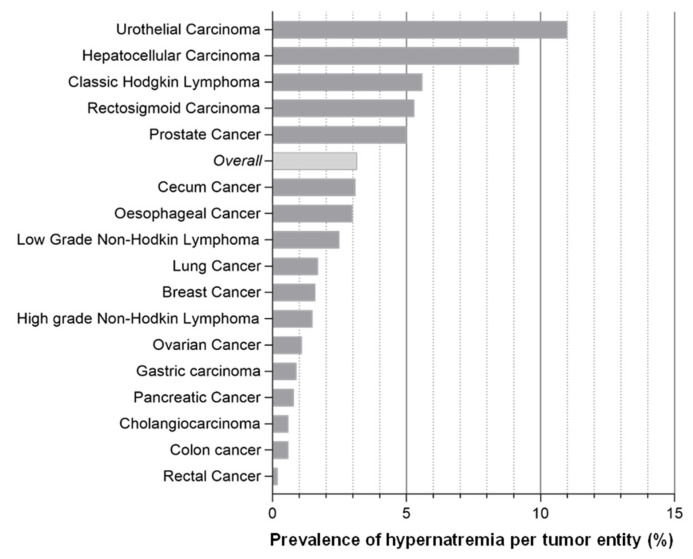
Prevalence of Hypernatremia according to Tumor Entity. Prevalence of hypernatremia in different tumor entities: The highest prevalence of 11% was found in patients with urothelial carcinoma. Patients with rectal carcinoma showed the smallest prevalence of becoming hypernatremic, with 0.2%.

Using a list of all oncology inpatients from the investigation period, we were able to determine the frequency of hypernatremia for each tumor entity. Among the 2945 patients treated in the oncology ward during the study period, 3.16% were hypernatremic at least once. The highest prevalence (11%) of all hypernatremia cases was found in patients with urothelial cancer. The least risk for hypernatremia was seen in patients with rectal carcinoma (0.2%) (Figure 3).

### 3.4. Survival According to the Onset and Reversibility of Hypernatremia

**Figure 4:**
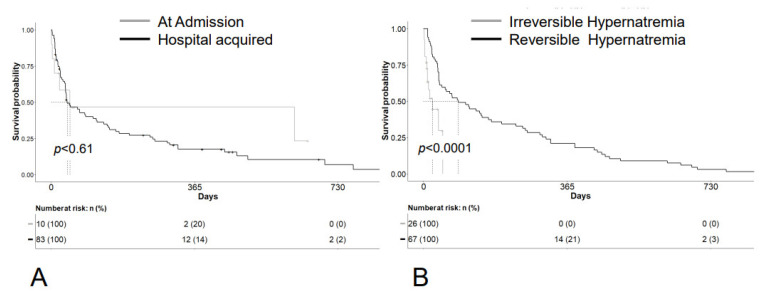
Survival According to the First Occurrence and Reversibility of Hypernatremia. (**A**) Overall survival according to the first occurrence of hypernatremia: Patients from the hypernatremic group were divided into two subgroups (hypernatremia on admission (*n* = 10) versus hospital-acquired hypernatremia (*n* = 83)). Log rank test was performed to compare the two survival curves. Median survival was 48 days for the patients with hypernatremia on admission versus 41 days for the patients with in-hospital acquired hypernatremia and showed no significant difference (*p* = 0.61). (**B**) Overall survival according to the reversibility of the hypernatremia: Patients from the hypernatremic group were divided into two subgroups (irreversible hypernatremia (*n* = 26) versus reversible hypernatremia (*n* = 67)). Log rank test was performed to compare the two survival curves. Median survival was 23 days for the patients with irreversible versus 88 days with reversible hypernatremia and showed a highly significant difference (*p* < 0.0001).

We wanted to find out whether the timing of the onset of hypernatremia had an impact on patient survival. In the hypernatremia group, we included both patients who already had hypernatremia on admission (*n* = 10) and those who acquired the hypernatremia during the course of their hospitalization (*n* = 83). The median survival was not significantly different between these two subgroups (HR 1.23, 95% CI 0.56–2.67, *p* = 0.606). Patients with preexisting hypernatremia had a median survival of 48 days, and patients with hospital-acquired hypernatremia had a median survival of 41 days (*p* = 0.61) (Figure 4A). In the latter group, 66.7% of patients were hypernatremic within the first 7 days and 78% within 10 days. The remaining 22% developed hypernatremia between the 11th and 44th day (Figure S2).

We speculated that irreversible hypernatremia would have a much worse prognosis than reversible hypernatremia. To this aim, we analyzed the prognosis of hypernatremic patients by the reversibility of their hypernatremia. The median OS of patients with reversible hypernatremia (*n* = 67) was 88 days compared to 23 days for the patients with irreversible hypernatremia (*n* = 26) (HR 0.25, 95% CI 0.13–049, *p* < 0.0001). We also analyzed the median sodium value for each group which was 146 mmol/L (range 146–160 mmol/L) for the group with reversible and 147.5 mmol/L (range 146–152 mmol/L) for the group with irreversible hypernatremia (*p* = 0.42) (Figure 4B).

### 3.5. Length of Hospital Stay and Discharge Status

**Figure 5:**
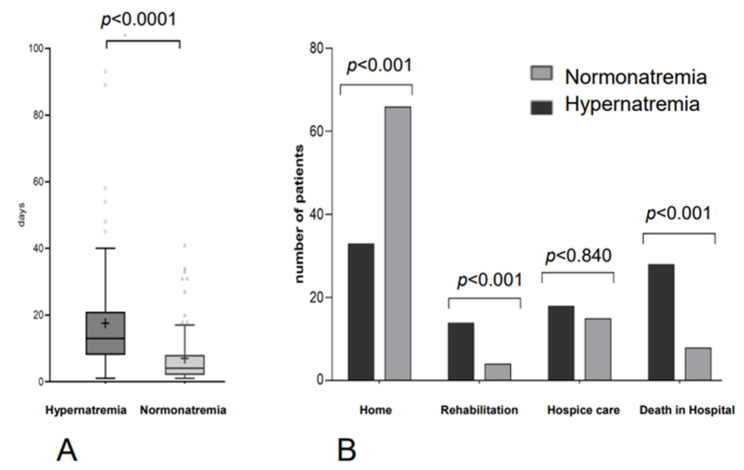
Length of Hospital Stay and Functional Status at Discharge. (**A**) Comparison of the length of hospital stay between the two groups: Length of hospital stay was defined as the time from the day of admission until the day of discharge or death. (**B**) Comparison of the number of patients according to status at discharge: The two groups differ significantly in the number of deaths, patients who went to rehabilitation or hospice-care clinics, and those who were discharged home after the hospital stay.

We wanted to evaluate whether hypernatremic patients have longer hospitalization times due to their electrolyte disturbance than normonatremic patients. For this purpose, we determined the duration of hospitalization, which was measured from the day of admission until the day of hospital discharge (length of hospital stay). The length of hospital stay for hypernatremic patients ranged from 1 to 93 days, and for normonatremic patients, from 1 to 41 days. The median time to discharge was significantly higher for the hypernatremic group with 13 days compared to 4 days for the normonatremic group (HR 1.25, 95% CI 0.54–2.91, *p* < 0.0001) (Figure 5A).

To determine the in-hospital mortality and the performance at the end of hospitalization on the oncology ward, we recorded the discharge status for each patient (discharge home, leaving for rehabilitation, leaving for hospice care, or death). The in-hospital mortality rate for the hypernatremia group was 30.1% and 8.6% for the normonatremic group (*p* < 0.001) (Figure 5B). The 90-day mortality rate for hypernatremic patients was 64.5% compared to 39.8% of normonatremic patients (*p* < 0.001). In total, 71% of the normonatremic patients and 35.5% of the hypernatremic patients were directly discharged home, while 4.3% of the normonatremic patients and 15.1% of the hypernatremic patients went to a rehabilitation clinic (Figure 5B).

## 4. Discussion

In our single-center retrospective study, hypernatremia was found in 3.16% of cancer patients in a general oncology ward. This low prevalence is well in line with previous similar studies describing a prevalence of 2–3% [5,11,13]. In ICU patients, the prevalence of hypernatremia was described to be higher at 9% [14]. Well known is the occurrence of hypernatremia in neurological critical care with an incidence of 8.5% [15], especially after brain tumor operations, e.g., craniopharyngiomas [16]. This high prevalence is well explained by the direct involvement of the neuroendocrine axis regulating the water balance. 

Our findings with respect to prognosis support our main hypothesis: cancer patients with hypernatremia have a significantly worse prognosis compared to normonatremic cancer patients (HR 0.38, 95% CI, 0.26–0.56, *p* < 0.0001; median OS 1.5 versus 11.5 months). Our results are in accordance with the findings of previous similar studies [5,15] and provide additional new evidence for the specific group of cancer patients admitted to a general oncology ward. Salahudeen et al. described a worse outcome with increased in-hospital mortality and 90-day mortality (47.2% versus 7.5%) in a hemato-oncological cancer center, including an intensive care unit and including patients with acute leukemias [5]. Seo et al. have investigated electrolyte disturbances in terminally ill patients on admission to a hospice care unit [11]. While the median overall survival time of all patients in the palliative care unit was 26 days (*n* = 487), it was only six days for hypernatremic patients (*n* = 15), markedly shorter than in our study. With a median overall survival of 1.5 months in our study, the prognosis of the hypernatremic cancer patients on the general oncology ward lies in between the prognosis described for the patients admitted to hospice care and the prognosis of the patients treated in the hemato-oncological cancer center including an intensive care unit. Overall, all three studies show the prognosis of cancer patients with hypernatremia to be very poor. Even if we cannot prove it formally, we assume that the differences in survival between the studies are most likely explained by the selection of the admitted patients rather than the quality of the treatment. Patients and management in our study are representative of contemporary in-patient recruitment and of current practices in a general oncology department. Whether differences in the oncological treatment before or after measurement of the sodium value contributed to the differences in the prognosis of our patients remains unclear.

We intended to explore the prognostic implication between preexisting hypernatremia on admission and acquired hypernatremia in the hospital. The number of patients with hypernatremia on admission in our study was small, with 11% (*n* = 10), similar to the study by Salahudeen et al. [5], suggesting that hypernatremia in our patients might frequently be of iatrogenic origin. There might be a decreased capability of these patients to regulate the salt-water balance. While other studies [9] showed a shorter survival in patients with hospital-acquired hypernatremia compared to patients with hypernatremia at admission, we could not observe a statistically significant difference and the numerical difference was small (41 versus 48 days).

In our study, 28% of the patients showed irreversible and 72% reversible hypernatremia. This rate is very similar to the study of Bataille et al. [17]. Our assumption of a worse prognosis in irreversible hypernatremia than in reversible hypernatremia was confirmed (23 days versus 88 days, *p* < 0.0001). One might assume that patients with irreversible hypernatremia had much higher sodium levels than patients with reversible hypernatremia. Even though the survival between the two groups was significantly different, the median sodium values did not exhibit a significant difference (p = 0.42): 147.5 mmol/L (range 146–152 mmol/L) and 146 mmol/L (range 146–160 mmol/L). These findings suggest that irreversibility is more important for the prognosis than the difference in the sodium concentration. The dismal prognosis is better explained with a fundamental underlying cause leading to an inability to precisely control the normally robust regulation of the salt-water balance rather than by the effects or even symptoms caused by the relatively small differences in sodium deregulation. 

One of the main limitations of our study was the retrospective, monocentric study design and the small sample size of hypernatremic patients due to the low prevalence of hypernatremia. Furthermore, it was not possible to retrospectively collect reliable information about the volume status of the patients and fluid losses such as diarrhea and vomiting to reliably explore the underlying pathophysiology of hypernatremia.

However, our study also has several strengths compared to other similar studies. First, the limitation of the small sample size of hypernatremic patients is outweighed by the matching procedure. Matching is a powerful and hypothesis-free tool to control for confounding factors. We have matched our cohorts for tumor entity, sex, and age. It is well described that age is an important risk factor for hypernatremia [18]. To our knowledge, our study is the first matched case-control study in hypernatremic cancer patients. Secondly, extracting the data from the hospital administrative and laboratory database helped to ensure the completeness of the dataset with the inclusion of all hypernatremic patients admitted over the observed period to minimize the risk of bias. This is supported by the finding of a prevalence of hypernatremic patients, which is very similar to other studies [5,15,17]. Thirdly with a median follow-up of 18 months, our study has a sufficient follow-up time to determine the mortality after discharge from the hospital and not only in-hospital mortality as in most other studies [5,15].

The very poor survival suggests hypernatremia serves as a prognostic parameter useful for decision making in the further therapeutic strategy: Antineoplastic therapy or switch to best supportive care in patients with a dismal prognosis. This would be to the advantage of this patient population and, incidentally, cost-efficient. Early in the disease, survival mostly depends on the stage of cancer. The more advanced a tumor, the more complex becomes the prognosis, with a simultaneous increase in its relevance. To predict survival in this setting, multiple previously described factors such as ECOG performance status, co-morbidities, complications, delirium, dyspnea, or anorexia-cachexia have to be taken into consideration [19]. Clinicians often base their therapeutic strategy on personal knowledge, clinical experience, and intuition which is known to be imprecise and overoptimistic [20]. Since no single prognostic factor proved to be very reliable and helpful on its own prognostic scores for a more objective estimate of the situation are in development [21]. Whether hypernatremia represents a factor that is independent of other prognostic factors could not be answered in our study since data on known competing prognostic factors, such as the ECOG performance status or the subjective impression of the treating physician, could only be incompletely collected in the retrospective setting. This obvious and simple subjective impression would need to be outperformed by a new prognostic marker.

However, for the few patients with an occurrence of hypernatremia, our data seem convincing that hypernatremia must be taken as a warning sign for a high risk of an unfavorable outcome. During observation over time, the irreversibility of hypernatremia is a further indicator of an even more unfavorable prognosis.

## 5. Conclusions

Hypernatremia in hospitalized patients on the oncology ward is associated with a high risk of a disadvantageous outcome with a very short median overall survival of only six weeks. In a few patients with the occurrence of hypernatremia, it can be used as a warning sign and an additional helpful decision tool for healthcare professionals to plan future treatment strategies. 

## Figures and Tables

**Table 1:** All patients were treated in the oncology ward at St. Claraspital between 2017 and 2020. Due to the matching criteria, in both groups were the same number of female and male patients, and the age was balanced.

	Hypernatremic Group *n* = 93	Normonatremic Group *n* = 93	*p*-Value
Sex			
Male (%)	52 (56)	52 (56)	-
Female (%)	41 (44)	41 (44)	-
Age [years] mean ± SD			
Mean	76.12 ± 8.95	75.61 ± 8.75	0.698
range	46.15–93.33	43.21–90.87	
Sodium Level [mmol/L]			
Range	146–160	135–145	-
Duration of hypernatremia [d]			
Mean SD	3 ± 2		-
Range	1–11		-
Length of hospital stay [d]			<0.0001
Mean SD	17.53 ± 15.96	6.98 ± 8.28	
Range	1–93	1–41	

## Data Availability

All data that support the findings of this study are included in this article. Further inquiries can be directed to the corresponding author.

## Supplementary Materials

The following supporting information can be downloaded at: https://www.mdpi.com/article/10.3390/curroncol29110693/s1, Figure S1: Types of Cancer in the Study Population; Figure S2: First Occurrence of Hypernatremia; Table S1: Co-morbidities; Table S2: Number of Co-morbidities and Number of Patients per Co-morbidity and Group; Table S3: Distribution of Tumor Stages.
