# Supplementary material for: Outcome of Hospitalized Cancer Patients with Hypernatremia: A Retrospective Case-Control Study
Source: Curr Oncol. 2022 Nov 16;29(11):8814–24. doi: 10.3390/curroncol29110693 (PMC9689174; doi:10.3390/curroncol29110693)
Supplement: Supplementary file 1 [file curroncol-29-00693-s001.zip › curroncol-2005847-supplementary.pdf]

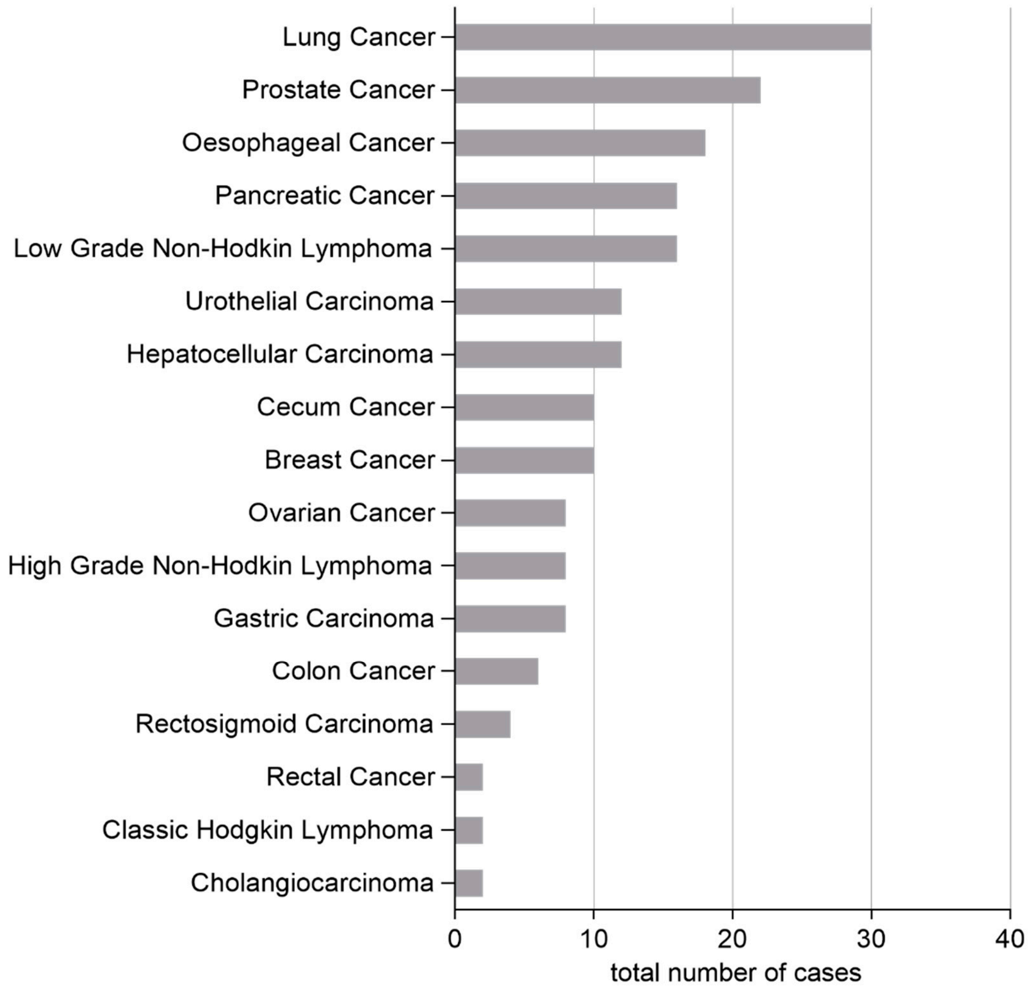

Figure S1. Types of Cancer in the Study Population.

Distribution of the oncological diagnosis in the two groups. Due to the matching process with the entity of the primary malignant disease as the main matching criteria, the number of patients per type of cancer in each group is the same.

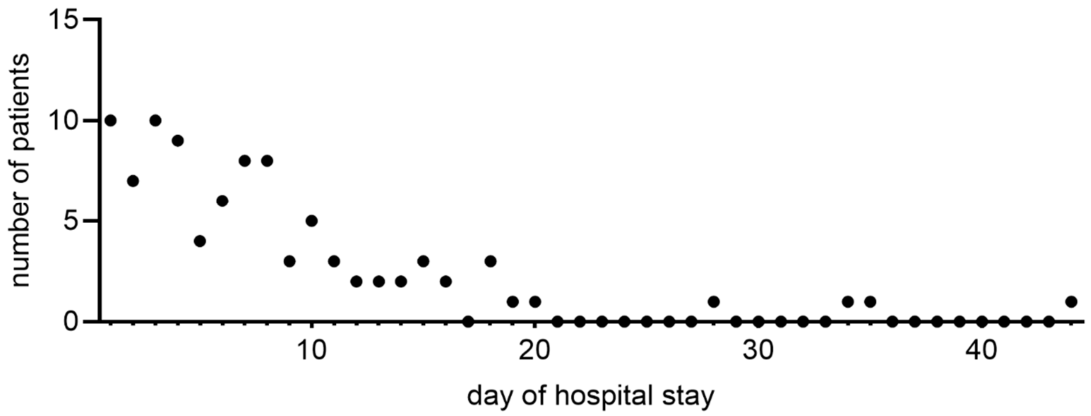

Figure S2. First Occurrence of Hypernatremia.

Overview of the first onset of hypernatremia for each patient: most patients were either hypernatremic at admission or became hypernatremic during the first few days of hospitalization.

Table S1. Co-morbidities.

Cardiovascular:	Heart failure, structural heart disease, coronary artery disease, previous heart attacks, previous strokes, peripheral artery disease, thrombosis, lung embolisms, or high blood pressure
Pulmonary:	Asthma, chronic obstructive pulmonary disease, emphysema, intestinal lung disease, previous lobectomy, or pneumectomy
Endocrinological:	Diabetes mellitus type I, II, or thyroid disease
Gastrointestinal:	Crohn's disease, ulcerative colitis, gastroduodenal ulcers, or active helicobacter pylori infection
Psychological:	Treatment required disease such as generalized anxiety disorder, depression, schizophrenia, bipolar disorder, or borderline personality disorders
Renal:	Acute and chronic kidney disease, previous nephrectomy, polycystic kidney disease
Neurological:	Parkinson’s disease, multiple sclerosis, central vertigo-associated disorders, Alzheimer's disease, epilepsy or epileptic syndrome
Previous Malignancies:	Other current stable malignancies or a previous solid tumor entity

Table S2. Number of Co-morbidities and Number of Patients per Co-morbidity and Group.

Number of Co-morbidities	Hypernatremia Group	Normonatremia Group	p-Value
	n = 93	n = 93	
0	1	9	0.023
1	10	25	0.009
2	27	19	0.234
3	27	21	0.402
4	16	16	-
5	9	2	0.062
6	3	1	0.613
**Relevant Comorbidities**	**Hypernatremia Group**	**Normonatremia Group**	**p-Value**
	n = 93	n = 93	
Cardiovascular	80	61	0.001
Pulmonary	23	15	0.146
Endocrinological	41	28	0.069
Gastrointestinal	27	19	0.174
Psychological	6	9	0.419
Renal	46	25	0.002
Neurological	13	13	-
Previous malignancies	18	33	0.014

Comparing the number of co-morbidities between the hypernatremia and the normonatremia group showed that in the hypernatremia group, most patients had two or three co-morbidities, whereas in the normonatremia group, it was one. The two groups differ significantly in the category without (p=0.023) or with one (p=0.09) co-morbidity.

Up to nine relevant co-morbidities were recorded for each patient. There is a significant difference in the number of co-morbidities between the two groups when it comes to cardiovascular disease (p=0.001), kidney disease (p=0.002), and previous malignancies (p=0.014).

Table S3. Distribution of Tumor Stages.

**Stage of the underlying malignant disease**	**Hypernatremia Group**	**Normonatremia Group**	**p-Value**
	n = 93	n = 93	
Lower stages: I and II *(%)*	5 *(5)*	19 *(20)*	0.004
Higher stages: III and IV *(%)*	77 *(83)*	62 *(67)*	0.018
NA *(%)*	11 *(12)*	10 *(11)*	-

Tumor stages were recorded according to the UICC TNM classification and were compared between the two groups. For both lower and higher tumor stages, the two groups were significantly different. Most patients (83% vs. 67%) showed a higher tumor stage (≥ III).
